# Diagnosis of spotted fever group *Rickettsia* infections: the Asian perspective

**DOI:** 10.1017/S0950268819001390

**Published:** 2019-10-07

**Authors:** Matthew T. Robinson, Jaruwan Satjanadumrong, Tom Hughes, John Stenos, Stuart D. Blacksell

**Affiliations:** 1 Lao-Oxford-Mahosot Hospital-Wellcome Trust Research Unit (LOMWRU), Mahosot Hospital, Vientiane, Lao People's Democratic Republic; 2 Nuffield Department of Clinical Medicine, Centre for Tropical Medicine and Global Health, University of Oxford, Churchill Hospital, Oxford, OX3 7FZ, UK; 3 Mahidol-Oxford Tropical Medicine Research Unit, Faculty of Tropical Medicine, Mahidol University, 420/6 Rajvithee Road, 10400 Bangkok, Thailand; 4 EcoHealth Alliance, 460 West 34th Street, 17th floor, New York, NY, USA; 5 Australian Rickettsial Reference Laboratory, Barwon Health, Geelong VIC 3220, Australia

**Keywords:** Asia, diagnosis, rickettsial infection, SFG, spotted fever *rickettsia*

## Abstract

Spotted fever group rickettsiae (SFG) are a neglected group of bacteria, belonging to the genus *Rickettsia*, that represent a large number of new and emerging infectious diseases with a worldwide distribution. The diseases are zoonotic and are transmitted by arthropod vectors, mainly ticks, fleas and mites, to hosts such as wild animals. Domesticated animals and humans are accidental hosts. In Asia, local people in endemic areas as well as travellers to these regions are at high risk of infection. In this review we compare SFG molecular and serological diagnostic methods and discuss their limitations. While there is a large range of molecular diagnostics and serological assays, both approaches have limitations and a positive result is dependent on the timing of sample collection. There is an increasing need for less expensive and easy-to-use diagnostic tests. However, despite many tests being available, their lack of suitability for use in resource-limited regions is of concern, as many require technical expertise, expensive equipment and reagents. In addition, many existing diagnostic tests still require rigorous validation in the regions and populations where these tests may be used, in particular to establish coherent and worthwhile cut-offs. It is likely that the best strategy is to use a real-time quantitative polymerase chain reaction (qPCR) and immunofluorescence assay in tandem. If the specimen is collected early enough in the infection there will be no antibodies but there will be a greater chance of a PCR positive result. Conversely, when there are detectable antibodies it is less likely that there will be a positive PCR result. It is therefore extremely important that a complete medical history is provided especially the number of days of fever prior to sample collection. More effort is required to develop and validate SFG diagnostics and those of other rickettsial infections.

## Introduction

Rickettsioses are infectious diseases caused by obligate intracellular gram-negative bacteria. They belong to the order Rickettsiales and reside in a wide range of arthropod vectors such as fleas, ticks and mites [1]. These vectors can transmit pathogens to humans at the bite site, who may or may not subsequently develop disease. Rickettsial diseases have been reported to be the second most common cause of non-malarial febrile illness in the Southeast Asia region after dengue infection [2].

Pathogenic members of the *Rickettsia* are classified into two major groups: spotted fever group (SFG) and typhus group (TG) rickettsiae and additional, *Orientia tsutsugamushi* and *O. chuto* are classified as scrub typhus group (STG) [3]. Although rickettsial diseases have worldwide distribution, there are endemic and hyper-endemic areas. TG and STG rickettsiae are widely diagnosed in Southeast Asia [1, 4, 5]. In Asia, TG infections are mainly caused by *Rickettsia typhi* [6, 7] which is the etiologic agent of murine typhus (or endemic typhus). *Rickettsia prowazekii*, also a TG rickettsiae and responsible for louse-borne typhus (epidemic typhus), is rarely detected. Scrub typhus is caused by *O. tsutsugamushi* along with the related *O. chuto* [8, 9]. *O. tsutsugamushi* is widespread in the Asia-Pacific (and northern Australia) however, when *O. chuto* is included, the geographical range is extended to include the Middle-east, Africa and South America [9, 10]. The SFG consists of over 30 species that can be found worldwide. The one of the most studied is *R. rickettsii* which causes Rocky Mountain spotted fever (RMSF) in North America [11]. Other species such as *R. australis* and *R. honei* are prevalent in northern Australia [12]. *Rickettsia conorii* is responsible for Mediterranean spotted fever (MSF) in several parts of Europe, Africa and Asia [13–15]. *Rickettsia felis* (known as the causative agent of flea-borne spotted fever) is seen as an emerging infectious disease. First identified in the USA and now seen worldwide, it is also responsible for many cases of febrile illness in Africa [16].

The main arthropod vectors of SFG are hard ticks (*Ixodidae*), although soft ticks (*Argasidae*) are also implicated in a number of SFG [1, 17]. Other SFG such as *R. felis*, may be transmitted by fleas, whilst more recently, mosquitoes have also been demonstrated to be competent vectors [18]. Dependent on the vector, transmission of SFG occurs via salivary products produced during feeding or through inoculation with the faeces of infected vectors on the wound, mucosal surfaces and via inhalation. Rickettsial infections occur following infection of the endothelial cell lining of blood vessels (microvascular endothelium in the case of infection by *R. conorii* and both microvascular and macrovascular endothelium in the case of *R. rickettsii*) [19, 20]. Symptoms at clinical presentation are variable and are often similar to many other acute febrile illnesses. Careful clinical observation by physicians and reliable laboratory diagnosis can lead to early appropriate administration of antibiotic therapy and patient management and thereby reduce patient mortality or clinical complications. The purpose of this article is to compare and contrast molecular and serological identification methods including limitations for the diagnosis of SFG infections with reference to the Asian perspective especially in low- and middle-income countries (LMICs)

## SFG infection and diagnosis

### Infection and clinical presentation

**Fig. 1.**
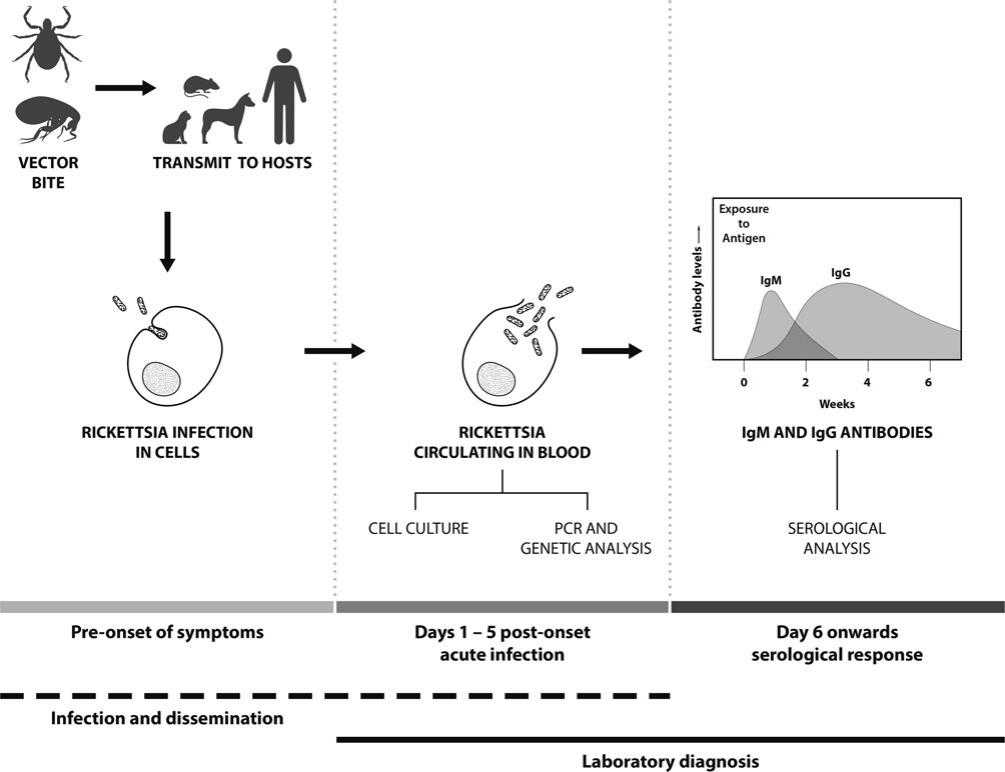
Features and temporal aspects of SFG *Rickettsia* infection and diagnosis.

The infection cycle of SFG in humans starts with the arthropod bite (normally ticks or fleas) followed by an incubation period of up to fifteen days prior to the onset of clinical symptoms (Fig. 1). Clinical symptoms of SFG infection can vary, ranging from mild to life-threatening. Patients suspected of SFG infection normally present with fever, nausea, vomiting, maculopapular rash and occasionally eschars at the site of inoculation [21]. SFG infection can result in variable and often severe clinical symptoms in individual patients and the lack of eschar evidence can lead to misdiagnosis and often delays in commencing antibiotic treatment. For instance, in Hong Kong, treatment was delayed due to misdiagnosis of a case of MSF infection with meningitis symptoms; this resulted in inappropriate treatment and a fatal outcome [22]. In addition, some MSF patients have shown complications such as hearing loss [23], acute myocarditis [24] and cerebral infarction [25]. In Japan, severe Japanese spotted fever cases (*R. japonica*) have been reported with acute respiratory failure complications, including acute respiratory distress syndrome [26, 27]. In other cases, complications from SFG infections can include renal failure, purpura fulminans and severe pneumonia [28]. Therefore, the availability of diagnostic techniques for SFG infections is important, as well as SFG-awareness of physicians to enable the early administration of appropriate antibiotic treatment.

### SFG laboratory diagnosis

#### Molecular detection

Polymerase chain reaction (PCR)-based detection is the primary method to detect SFG, especially for the early detection of infection before the development of detectable antibodies [29] (Fig. 1). Molecular-based techniques need to be sensitive, and the sample type and assay used determine the success of detection. PCRs are used for both epidemiological and diagnostic purposes and SFG DNA may be isolated from arthropods, animal hosts and human clinical samples including whole blood, buffy coat, serum, tissue biopsies (such as skin), eschar scrapings and swabs [30]. For human clinical diagnostic purposes, whole blood or buffy coat is the preferred sample type, as SFG are intracellular (the cellular component being concentrated in the buffy coat fraction, increasing the sensitivity of detection) and the sample type being easily collected. Concentration of the sample type and maximizing the sensitivity of assays are a very important consideration for the detection of rickettsiae. Although little is known of the quantities of SFG in blood, quantities of other rickettsial organisms in the blood of an infected patient are variable. In RMSF with nonfatal outcomes, *R. rickettsii* copy numbers ranged from 8.40 × 10^1^ to 3.95 × 10^5^ copies/ml of blood, whilst in patients with fatal infections copy numbers ranged from 1.41 × 10^3^ to 2.05 × 10^6^ copies/ml of blood [31]. By comparison, the STG *O. tsutsugamushi* may be found at a median density of 13 genome copies/ml of blood (IQR: 0–334) [32]. Serum or plasma may be used for the PCR, but these samples are less than optimal as there will be fewer patient cells (meaning lower rickettsiae concentration). In addition, serum may have increased concentrations of blood fibrinogen and fibrin materials which can bind to *Rickettsia* DNA decreasing availability of DNA target for PCRs [33]. Eschars are a suitable sample type for the PCR (as well as culture techniques) and may be sampled as either scrapings, swabs or biopsied specimen [34]. As with any sample type, the specimen needs to be maintained at optimal temperatures or preserved prior to detection and/or isolation. Formalin-fixed and paraffin-embedded tissues can also be used, but fixation using formalin causes nucleic acid fragmentation and reduces the quality and quantity of nucleic acids as well as limiting the length of PCR products [35].

Selection of an appropriate gene target is important. Conserved gene targets enable a broad *Rickettsia* genus- or SFG-level of detection. The use of gene targets such as the citrate synthase (*gltA*), 16S rRNA and 17 kDa lipoprotein outer membrane antigens (17 kDa) generally confirms the presence of SFG or TG rickettsiae [36–39]. The use of Outer Membrane Protein A (190 kDa) (*ompA*) and B (*ompB*) genes appear to be more specific and discriminating for SFG in both patient and animal samples [29, 38].With all of these gene targets, down-stream sequencing of the PCR product will discern, in most cases, specific species. It is strongly recommended that multiple gene targets are used to gain an accurate identification.

A variety of different PCR-based assays are available for the diagnosis of SFG infections. Table 1 lists the most commonly used conventional (cPCR), nested (nPCR) and quantitative PCRs (qPCRs) that have been developed. Many of the primer sets have been mixed-and-matched and optimized in different studies to maximize the identification of SFG infections. cPCR assays have targeted most of the key genes discussed. nPCRs (which use two sets of primers) have been developed to increase the detection sensitivity over cPCRs although, as with other PCR assays, there may still be difficulty in differentiating closely-related SFG species, such as *R. conorii* and *R. sibirica* [40]. Several real-time or quantitative PCR (qPCR) assays developed for rickettsial detection have largely replaced the use of nPCRs due to a greater sensitivity and shorter run-time (Table 1). Furthermore, multiplexed qPCR is an efficient method that demonstrates greater analytical power, as multiple primers and probes are combined into a single assay, either targeting a number of different species or different gene targets for a single species at the same time [35].

**Table 1.** Summary of common SFG PCRs used for diagnosis and sequencing for species identification purposes

Target	Oligo name	Oligo combinations									PCR type	Original reference	PCR use	Further references
16S rDNA	16SU17F^a^					1	2				(1) cPCR (2) nPCR	(1) [41] (2) [42]	VD/HD/S	[29]
	Rc16S.452n					1	–							
	16SU1592R					–	2							
	16sR34F					–	2							
	16sOR1198R					–	2							
17 kDa	R17K249R					1					(1) qPCR^b^	[43]	VD/HD	
	R17K135F					1								
	R17Kbprobe					1								
	R17K128F2					1					(1) qPCR^c^	[44]	VD	[42,45, 46]
	R17K238R					1								
	R17K202TaqP					1								
	R17–122			1	–	3	4				(1) cPCR (2) cPCR (3) nPCR (4) nPCR	(1/4) [47] (2) [48]	HD	[11, 38, 49]
	R17–500			1	–	3	4							
	Tz-16–20			–	2	3	–							
	Tz-15–19			–	2	3	–							
	RP1D			–	–	–	4							
	RP2			–	–	–	4							
	R17kM61F				–	–	3				(1) cPCR (2) nPCR (3) nPCR	[42]	VD/HD/S	[29, 45, 50]
	R17k31F^d^				1	2	3							
	R17k469R				–	2	3							
	Rr17k2608RN^e^				1	2	–							
*gltA*	CS1dF					1	2				(1) cPCR (2) nPCR	(1) [51] (2) [52]	VD/S	[45]
	CS1273R					1	2							
	CS1234R					–	2							
	RpECS1258n^f^					1	2				(1) cPCR (2) nPCR	(1) [51, 53] (2) [40]	VD/HD/S	[29, 38, 45, 49]
	RpCS.877 p^g^					1	2							
	RpCS.1233n					–	2							
	RpCS.896p					–	2							
	CS-78					1					(1) cPCR	[54]	VD/HD	[29]
	CS-323					1								
	CS-F					1					(1) qPCR	[55]	VD/HD	[42]
	CS-R					1								
	CS-P					1								
*ompA*	RompA642R					1					(1) cPCR	[42]	VD/S	[45]
	RompAM50F					1								
	RR190-70	1	2	3	4	–	–	–	–	–	(1) cPCR (2) cPCR (3) nPCR (4) cPCR (5) nPCR (6) cPCR (7) nPCR (8) cPCR (9) qPCR	(1) [49] (2) [53] (4) [56] (7) [36] (8) [57]	VD/HD/S	[11, 29, 45, 58]
	RR190–701	1	−	3	4	5	6	–	–	9				
	190-RN1	1	–	–	–	–	–	–	–	–				
	190-FN1	1	–	–	–	–	–	–	–	–				
	Rr190k.720n	–	–	–	–	–	–	7	–	–				
	Rr190k.71p	–	–	–	–	–	–	7	–	–				
	RR190–602	–	2	3	–	–	6	7	–	–				
	RR190.547F	–	–	–	–	5	–	–	8	9				
	RR190.701R	–	–	−	–	5	–	–	8	–				
*ompB*	Rf1524R					1					(1) cPCR	[42]	VD/S	
	ompB1570R					1								
	120–607F					1					(1) nPCR	[39, 59] (#237)	VD/S	[45]
	RompB11F					1								
	RompB1902R					1								
	RAK1009F					1					(1) cPCR	[59] (#237)	VD/S	[45]
	RAK1452R					1								
	Rc.rompB.4836n					1					(1) nPCR	[40]	HD	[38]
	Rc.rompB.4362p					1								
	Rc.rompB.4762n					1								
	Rc.rompB.4496p					1								
	rompB OF					1					(1) nPCR	[40]	VD/HD	[29]
	rompB OR					1								
	rompB SFG IF					1								
	rompB SFG IR					1								
*rplP*	PanR8-F					1					(1) qPCR	[11]	HD	
	PanR8-P					1								
	PanR8-R					1								
*sca4*	RrD928R					1					(1) nPCR	[59](#237)	VD/S	[45]
	RrD1826R					1								
	RrD749F					1								

Species-specific PCRs excluded. PCR type: cPCR, conventional; nPCR, nested (including semi-nested); qPCR, quantitative/real-time PCR. PCR use: VD, diagnostics in arthropod vectors; HD, diagnostics in human samples; S, used for sequencing purposes.

a aka: fD1.

b aka: Rick17 assay.

c aka: Rick17b assay (developed from Rick17 assay).

d aka: 17 kDa-1/Primer1; addition of ACA on 3′ end in references [42,45].

e aka: 17 kDa-2/Rr2608Rnew/Primer2; base change G20A in references [29,42,45]; base change T7C in references [42,45]; base change A22C in reference [38].

f Base change A22C in reference [38].

g aka: CS1273R; base change G5A in reference [38].

An alternative to the standard PCR assays, suitable for more field-based diagnosis, is the loop-mediated isothermal amplification assay (LAMP). This has a potential for application as a simple and rapid molecular SFG detection technique, employing an isothermal (constant temperature) nucleic acid technique for the amplification of DNA. The amplification is performed at 60–65 °C and the stop reaction at 80 °C [60]. The LAMP has been reported to be more sensitive than the nPCR to detect *ompB* from SFG in China, with 73% sensitivity and 100% specificity compared with the nPCR which gave negative results [60].

As with all PCR assays, it is also possible that a vector may carry more than one rickettsial species resulting in multiple PCR products being obtained if using broad range primers (not species-specific). This can cause difficulties in interpreting results, and may even result in misdiagnosing of the actual agent causing disease. Therefore, if the PCR results show positivity for SFG but the assay cannot differentiate between species, the amplicons should be sequenced for further species identification which provides useful epidemiological information.

### Serological detection

The genus *Rickettsia* and *Orientia* can be characterized into three major antigenic groups: SFG, TG and STG [37]. There are serological cross reactions between SFG and TG antibodies [17, 30, 37] as well amongst antibodies of *Rickettsia* spp. within SFG and therefore serological diagnosis is normally only made to the antigenic (serogroup) group level. Discrimination to the species level within SFG using serological techniques is extremely difficult and only possible following cross-absorption using western blot techniques [30, 61].

In many cases, rickettsial infection is confirmed by the use of serological techniques such as indirect immunofluorescence assay (IFA), indirect immunoperoxidase (IIP) test, enzyme-linked immunosorbent assay (ELISA) and the Weil–Felix agglutination test (WFT). Immunoglobulin M (IgM) antibodies are present in the early phase of an infection and reduces afterwards until they are undetectable after a few weeks (Fig. 1). In contrast, IgG increases after the second week of illness and persists at low titers for years in some cases (30% found after one year) [62]. The WFT was developed in 1916 and primarily used in mid-1940's for the identification of TG rickettsiae infections [63]. The WFT is an inexpensive test, principally based on the antibodies against Gram-negative *Proteus* spp. antigens of OX (O-specific polysaccharide chain of outer membrane lipopolysaccharide) which cross-reacts with *Rickettsia* antigens OX-2 and OX-19 with the former more specific to SFG [64]. However, the WFT demonstrates low sensitivity in the acute phase of infection as demonstrated by 47% sensitivity in RMSF [65] and 33% sensitivity for SFG in Sri Lanka [66] and is rarely use nowadays to this shortcoming.

In many Asian countries, IFA and IIP are recognized as standard methods for routine diagnosis. The sensitivity of IIP and IFA often depends on the timing of serum collection due to the lack of antibodies in the first week of illness prior to complete seroconversion [67] although it can demonstrate high sensitivity (83–100%) and specificity (99–100%) (Table 2) [37]. A seroconversion or a four-fold difference in antibodies from acute to convalescent phase IgM and IgG antibodies is considered to be significant [62, 68] however, the diagnostic accuracy of such tests is also dependent on the cut-offs applied and therefore a understanding of the background immunity in endemic and non-endemic populations is essential with higher cut-offs often used in endemic settings. Furthermore, IFA and IIP results are dependent on appropriate antigenic types (often *R. honei* and *R. conorii* in Asia), well-trained personnel for the interpretation of the results, and requires a fluorescence microscope, which is often expensive and difficult to maintain.

**Table 2.** The comparison of sensitivity and specificity of serological techniques for SFG infection

Year	Location	Method	Sample type	Samples	% Sens	% Spec	Rickettsial species	Reference
1975	USA	IFA	Serum	571	85–97	99–100	RMSF	[69]
1975	USA	IFA	Serum	42	86	NA	SFG	[70]
1983	USA	IFA-IgG/M	Serum	50	83–100	100	RMSF	[71]
1981–1984	Georgia	IFA	Serum	1774	94	NA	RMSF	[65]
1983	USA	ELISA-IgG ELISA-IgM	Serum	50	93–100 100	100 100	RMSF	[71]
2009	India	ELISA-IgM	Blood	161	91	100	SFG & STG	[72]
2010–2012	India	ELISA-IgG/M	Serum	103	85	100	SFG	[73]
1975	USA	CF	Serum	571	85.4	100	RMSF	[69]
1975	USA	CF	Serum	42	62	NA	SFG	[70]
1981–1984	Georgia	CF	Serum	1774	63	NA	RMSF	[65]
1975	USA	WF	Serum	42	21	NA	SFG	[70]
1981–1984	Georgia	WF (OX-19) WF (OX-2)	Serum	1774	70 47	NA NA	RMSF	[65]
2001	Sri Lanka	WF	Serum	30	33	46	SFG, STG & TG	[66]
2009	Central India	WF	Serum	157	49	96	SFG & STG	[72]
1975	USA	MA	Serum	571	56.1	99	RMSF	[69]

NA, not available; ST, scrub typhus; MT, murine typhus; Sens, sensitivity; Spec, specificity; WF, Weil–Felix; CF, complement fixation; IFA, immunofluorescence assay; RMSF, Rocky Mountain spotted fever; SFG, spotted fever group *Rickettsia;* STG, scrub typhus group; TG, typhus group.

Enzyme-linked immunosorbent assays (ELISAs) are widely used and demonstrate high sensitivity and specificity. In an Indian study, researchers used a commercial *R. conorii* ELISA IgG/IgM kit that demonstrated 85% sensitivity and 100% specificity [73] (Table 2). The advantage of ELISA methodologies is that they allow screening of large batches of samples, which is less time-consuming, and provide more objectivity compared to the subjective IFA, as there is no reader-bias due to the use of optical density (OD) by ELISA readers. A diagnostic cut-off OD of ⩾0.5 has been applied previously to the SFG ELISA developed by the US Naval Medical Research Center [74, 75], however, there is no independent validation of the use of this cut-off. However, ELISAs may not recognize all *Rickettsia* pathogens and may be negative at the acute stage of an infection depending on the isotype (IgM or IgG) (Fig. 1), and results can only be obtained after seroconversion (around 5–14-days post onset of illness) [71,72]. Collecting only a single sample from patients can often make diagnosis difficult [76] and it is recommended that both acute and convalescent samples are collected whereby a significant rise in antibody levels is then considered as indicative of an active infection.

### Need to improve diagnosis and clinical awareness of SFG

There is an urgent need to improve diagnosis and awareness of SFG in Asia. Generally, SFG infections are significantly neglected and under recognized in Asia. Spotted fever rickettsiae diseases often have non-specific clinical symptoms and may be overlooked by physicians [61, 77], although awareness is increasing with improved diagnostics [17, 33, 37] and publication of findings. SFG are also under recognized by laboratory staff as SFG are obligate intracellular bacteria whose culture *in vitro* is complicated, lengthy, expensive and often reserved to specialized laboratories equipped with BSL3 level containment facilities [78].

SFG may be misidentified as TG *Rickettsia* due to serological cross-reactions in which the r*OmpB* protein appears to play an important role [79–81]. Moreover, sequencing data indicates that some 50 amino acids in *rOmpB* of *R. japonica* (SFG) are identical to that of *R. typhi* (TG) [82]. Antibodies for *R. typhi* have demonstrated greater cross-reaction with *R. conorii* than *R. rickettsii* and higher cross-reactivity with IgM than IgG antibodies. However, there may be enough difference between end point titers to permit identity between TG and SFG [83]. The cross-reacting antibodies may also depend on the immunogenic responses of each patient [82] and multiple infections and re-infections also make it difficult to distinguish between species due to the broadness of the immune response.

In the case of molecular diagnostic tests, adoption of PCR technologies is limited due to cost, lack of expertise and technical issues. Although often highly sensitive and specific, PCR techniques have limitations as false-negative results may result from low quantities of SFG and the transient nature of these pathogens present in the circulating blood [84]. In reality, the major limiting factor for the use of PCR methods is the need for expensive thermocycler equipment, reagents and specific primers. Despite this, these techniques should be encouraged as PCR assays are highly useful, they allow accurate SFG identification, enable the discovery of novel species, are increasingly affordable, reproducible and less-time consuming with high specificity and sensitivity especially in the early phase of infection [85].

### Diagnostic cutoffs in seroprevalence studies

Seroprevalence studies provide important information regarding the distribution of SFG in community- or hospital-based settings. The choice of diagnostic cut off may greatly influence the seroprevalence results, with a low cut off often giving high sensitivity but low specificity, whereas a high cut off will give low sensitivity but high specificity [69]. Seroprevalence studies of SFG in Asia have used IFA, IIP and ELISA techniques (Table 1) with IFA considered to be the diagnostic gold standard for the quantitative detection of rickettsial antibodies [30]. However, there appears to be a lack of consistency in the application of the cut-off titer criteria depending on the geographic location (Table 2). In the case of seroprevalence studies, the application of a consistent regional cut off will more readily reflect true endemicity and enable comparability between countries and regions. In Southeast, South and East Asia, studies have used reasonably consistent cut-off titers of ⩾1:64 in Philippines [86], Sri Lanka [87], Thailand [3] and Bangladesh [88] and in South Korea and Taiwan, a cut-off of 1:40 is considered as positive [89, 90]. However, different cut-off values have been reported for IgM and IgG isotypes, such as ⩾1:128 for IgG and ⩾1:64 for IgM in Thailand [3]. Interestingly, Denmark has used a cutoff as high as 1:512 [79]. There is a need to standardize diagnostic cut-offs for seroprevalence studies to allow easier comparability of results.

## Conclusions

SFG *Rickettsia* causes a large number of emerging and re-emerging infectious diseases with a worldwide distribution. Its occurrence in LMICs exacerbates an already problematic diagnostic issue. With a limited range of suitable sensitive and specific tests available, the additional compounding factor of the need for cheap and easy to use diagnostic tests inputs additional burden on the populations exposed to these pathogens. Despite many tests being available, their lack of suitability for use in resource-limited regions is of concern, as many require technical expertise, expensive equipment and reagents. In addition, many existing diagnostic tests still require rigorous validation in the regions and populations where these tests may be used, in particular to establish coherent and worthwhile cut-offs. Possibly the best strategy would be to use a qPCR and IFA in tandem whereby, if the specimen is collected early enough in the infection where there will be no antibodies, there is a greater chance of a PCR-positive result. Conversely, if there are detectable antibodies, it is less likely that there will be a positive PCR result. We are in an age when more and more novel diagnostic tests are coming onto the market, and we need to ensure that these tests are suitable and appropriate for the diagnosis of rickettsial diseases, especially in low-income countries.
